# Analyzing the Opportunities to Target DNA Double-Strand Breaks Repair and Replicative Stress Responses to Improve Therapeutic Index of Colorectal Cancer

**DOI:** 10.3390/cancers13133130

**Published:** 2021-06-23

**Authors:** Paula Pellenz Tomasini, Temenouga Nikolova Guecheva, Natalia Motta Leguisamo, Sarah Péricart, Anne-Cécile Brunac, Jean Sébastien Hoffmann, Jenifer Saffi

**Affiliations:** 1 Laboratory of Genetic Toxicology, Federal University of Health Sciences of Porto Alegre, Avenida Sarmento Leite, 245, Porto Alegre 90050-170, Brazil; paula-tomasini@hotmail.com (P.P.T.); nmleguisamo@gmail.com (N.M.L.); 2 Post-Graduation Program in Cell and Molecular Biology, Federal University of Rio Grande do Sul, Avenida Bento Gonçalves, 9500, Porto Alegre 91501-970, Brazil; 3 Cardiology Institute of Rio Grande do Sul, University Foundation of Cardiology (IC-FUC), Porto Alegre 90620-000, Brazil; tgesheva@gmail.com; 4 Laboratoire D’Excellence Toulouse Cancer (TOUCAN), Laboratoire de Pathologie, Institut Universitaire du Cancer-Toulouse, Oncopole, 1 Avenue Irène-Joliot-Curie, 31059 Toulouse, France; pericart.sarah@iuct-oncopole.fr (S.P.); brunac.annececile@iuct-oncopole.fr (A.-C.B.); jean-sebastien.hoffmann@inserm.fr (J.S.H.)

**Keywords:** colorectal cancer, DNA double strand break repair, replication stress, target therapy

## Abstract

**Simple Summary:**

Colorectal cancer (CRC) is among the most common cancers and the third leading cause of cancer deaths worldwide. Despite the identification of alterations in DNA repair genes and the resulting genomic instability in sub-populations of CRC, therapies that exploit defects in DNA repair pathways or high level of replicative stress have been explored only in breast, ovarian, and other tumor types, but not yet systematically in CRC. Here, we discuss how targeting genes involved in the responses to replication stress and the repair of DNA double-strand breaks (DSBs) could provide new therapeutic opportunities to treat CRCs and have the potential to confer increased sensitivity to current chemotherapy regimens, thus, expanding the spectrum of therapy options, and potentially improving clinical outcomes for CRC patients.

**Abstract:**

Despite the ample improvements of CRC molecular landscape, the therapeutic options still rely on conventional chemotherapy-based regimens for early disease, and few targeted agents are recommended for clinical use in the metastatic setting. Moreover, the impact of cytotoxic, targeted agents, and immunotherapy combinations in the metastatic scenario is not fully satisfactory, especially the outcomes for patients who develop resistance to these treatments need to be improved. Here, we examine the opportunity to consider therapeutic agents targeting DNA repair and DNA replication stress response as strategies to exploit genetic or functional defects in the DNA damage response (DDR) pathways through synthetic lethal mechanisms, still not explored in CRC. These include the multiple actors involved in the repair of DNA double-strand breaks (DSBs) through homologous recombination (HR), classical non-homologous end joining (NHEJ), and microhomology-mediated end-joining (MMEJ), inhibitors of the base excision repair (BER) protein poly (ADP-ribose) polymerase (PARP), as well as inhibitors of the DNA damage kinases ataxia-telangiectasia and Rad3 related (ATR), CHK1, WEE1, and ataxia-telangiectasia mutated (ATM). We also review the biomarkers that guide the use of these agents, and current clinical trials with targeted DDR therapies.

## 1. Introduction

### 1.1. Standard of Care of Colorectal Cancer

Despite advances in screening, surveillance, and identification of clinical and molecular features holding significant prognostic value, colorectal cancer (CRC) still ranks the second leading cause of cancer-related deaths worldwide. The 5-year relative survival rate for CRC ranges from 90% for those diagnosed with localized disease to 14% for those with metastatic disease [1]. By 2030, an increase of 60% in the CRC global burden to more than 2.2 million new cases is expected [2], with a worrisome incidence rate increase up to 124% for patients in the age group of 20 to 34 years [3].

Most colorectal tumors occur sporadically and result from a 10 to 15 years sequenced tumorigenic process comprising a persistent accumulation of mutations, also known as adenoma-carcinoma sequence [4,5]. CRC screening through colonoscopy is recommended beginning at age 50 years and then every 10 years in order to detect and remove precursor lesions or, at least, identify the disease in its earliest stages [6]. However, up to 25% of CRC cases are diagnosed when distant metastases are already present [7].

The tumor-node-metastasis (TNM) classification and traditional clinicopathological assessment are commonly used to determine disease prognosis and to drive therapeutic decision-making in CRC. Since survival outcomes between colon and rectum tumors according to the TNM stages are very similar, these diseases therefore share the same staging system [8]. Treatment of stage I CRC (T1-T2, N0, M0) is based on surgical removal and surveillance with colonoscopy, and adjuvant therapy is not recommended. Stages II (T3-T4b, N0, M0) and III (any T, N1–N2b, M0) comprise non-metastatic CRC. For resectable non-metastatic CRC, the preferred surgical procedure is colectomy with removal of the regional lymph nodes [9]. Systemic treatment regimens for stages II and III CRC include doublets or triplets of cytotoxic chemotherapy (5-fluorouracil—5FU or capecitabine, oxaliplatin, and irinotecan). Targeted therapies, including antiangiogenics (bevacizumab, ramucirumab, or ziv-aflibercept) and monoclonal antibodies anti-EGFR (cetuximab, panitumumab) or anti-BRAF (encorafenib) or immune checkpoint inhibitors (anti-PD1/PD-L1 and anti-CTLA4) are the options for metastatic CRC [10].

While for stage III CRC adjuvant systemic therapy is mandatory, stage II CRC must be firstly assessed for potential risk/benefits of therapy and prognosis. Currently, adjuvant chemotherapy for stage II CRC is recommended to patents with less than 12 lymph nodes analyzed after surgery and poor prognostic features (such as poorly differentiated histology, lymphatic/vascular invasion, positive margins, obstruction/perforation, comorbidities) [11]. Currently, the options of adjuvant therapy for high-risk stage II CRC are FOLFOX (5-fluorouracil, leucovorin, and oxaliplatin), CAPEOX (capecitabine and oxaliplatin), capecitabine, or 5-fluorouracil/leucovorin for 3–6 months. The choice between these regimens depends on the patient’s performance status, T stage, the number of high-risk features for recurrence, and patient/physician preferences. In brief, single-agent capecitabine or 5-fluoouracil/leucovorin (5-FU/LV) are indicated for T3 and no high risk-features, while for T3 at high-risk of recurrence or T4 (stage IIB/IIC), FOLFOX, or CAPEOX are the preferred options [12]. Recently, microsatellite instability (MSI) or DNA mismatch repair (MMR) status has been included in risk assessment in stage II disease. For microsatellite-stable (MSS) or MMR-proficient (pMMR) low-risk stage II disease, only observation or adjuvant 5-FU/LV without oxaliplatin is recommended [13,14]. Similarly, stage III CRC is classified into high (T4, N1–2 or any, N2) and low (T1–3, N1) risk of recurrence. In this setting, patients are always referred to adjuvant treatment with CAPEOX (capecitabine, oxaliplatin), FOLFOX or 5-FU/LV if oxaliplatin is not tolerated [15]. This results in a 5-year disease-free survival (DFS) of 63.6% vs. 49.0% in those who did not receive adjuvant chemotherapy [16].

Standardized staging and one-size-fits-all approaches for localized to locally advanced disease fail to identify and deliver the optimal care for a group of patients who will inevitably present a poor prognosis. Inevitably, distinct drug responses and inconsistent survival outcomes are not uncommon within a single stage, which is even more pronounced regarding stages II and III [17]. The existence of a survival paradox between stages IIB/IIC and IIIA (T1–2, N1, M0 or T1, N2, M0) is a widely known phenomenon in CRC patients, and numerous causes had been described to contribute to an inferior survival in IIB/IIC compared with that of stage IIIA [11]. One explanation might be the insufficient use of systemic adjuvant chemotherapy in stage II CRC, since it is part of the standard care of treatment used in stage III CRC [18]. Another inconsistency refers to the distinction between high and low risk in stage II disease, since a significant number of high-risk patients with pathological and or prognostic features of poor outcomes will not develop disease recurrence and vice-versa. Furthermore, there are no data supporting the predictive value of the risk features and response to chemotherapy. Unfortunately, it has been shown that irrespective of the risk of recurrence, 5-year DFS rate in stage II patients undergoing adjuvant treatment was 81.4% (vs. 79.3%) [16], and the improvement of overall survival does not exceed 5% [10,19].

CRC is a heterogeneous disease, but these inconsistent responses to therapy and clinical outcomes may be resolved through incorporation of tumor molecular features to current staging. More recently, the identification of predictive molecular biomarkers has become standard practice for CRC management. Currently, the workup for metastatic disease recommends the investigation of activating mutations in *KRAS* (exon 2, 3, or 4) or *NRAS* (exon 2, 3 or 4) and *BRAF* (V600E), as well MMR status [20]. Within the metastatic disease setting, the presence of *KRAS*/*NRAS* mutations provides resistance to cetuximab and panitumumab [21], while *BRAF^V600E^* mutation predicts response to anti-EGFR targeted therapies in combination with a BRAF inhibitor [22,23]. In patients with known wild type *RAS* and *BRAF*, HER2 testing for amplification or overexpression is recommended. Thus, 3% of all stage IV CRC are eligible for combined anti-HER2 therapies (trastuzumab plus pertuzumab or lapatinib) [24,25]. Finally, the frequency of stage IV MMR-deficient (dMMR) CRC is 3.5% to 5%, and predicts the response to immune checkpoint inhibitors in first and subsequent lines [26,27,28]. Although several clinicopathological and molecular features are known to be determinant on the disease prognosis, the majority of predictive biomarkers, which have reached clinical practice, are limited to metastatic setting.

### 1.2. Molecular Classification of CRC Tumors

While genetic susceptibility to CRC includes well-defined inherited syndromes—Lynch syndrome or hereditary nonpolyposis CRC (HNPCC), polymerase proofreading–associated polyposis (PPAP) caused by germline missense mutations affecting the proofreading activity of polymerases epsilon (POLE) and delta (POLD1) and *NTHL1*-and *MUTYH*-associated polyposis (NAP and MAP, respectively), sporadic CRC is highly heterogeneous on the genetic and gene regulatory levels [29,30,31,32]. Although MSI status and a few numbers of mutations have reached clinical practice due their role on disease prognosis, they are still insufficient to identify all patients with increased risk of recurrence and metastasis. Thus, in order to translate the intra- and inter-tumor heterogeneity, gene expression signatures have been extensively investigated.

Transcriptomic analyses showed the molecular heterogeneity of CRC and established a molecular classification into four consensus molecular subtypes (CMS1–4) [33,34,35]. CMS1 (“immune”) comprises 14% of CRC tumors and is associated with hypermutable characteristics, mutations within the *BRAF* gene, microsatellite instability (MSI) and strong immune activation. The “canonical” CMS2 (37% of total CRC tumors) is associated with mutated *TP53* gene, APC mutations, activated WNT and MYC signaling, chromosomal instability (CIN), and copy number alterations. The “metabolic” CMS3 (13% of tumors), is characterized by metabolic dysregulation and *KRAS* mutations. The “mesenchymal” CMS4 (23% of tumors) is characterized by upregulation of genes involved in the epithelial to mesenchymal transition (EMT), transforming growth factor-β activation and inflammatory microenvironment. In view of the extensive biological differences between these subtypes, the ability to respond to therapies may also be different for each subtype [36,37]. It is important to note that CMS4 tumors present downregulation of all DNA repair pathways, which is attributed to hypoxia and a stem-like phenotype [38]. CRC CMS4 subtype is often diagnosed in advanced stages. However, it has been also reported that stages II-III patients also present the poorest prognosis among CMS subtypes, mostly due to increased progression rates towards metastatic disease [39]. Although current recommendations for CRC adjuvant treatment include high-risk stage II and all stage III patients, the benefit of chemotherapy in the adjuvant setting for stage II is still a matter of debate [12]. Moreover, while for metastatic disease, CMS4 tumors are resistant to anti-EGFR therapies (irrespective of *KRAS* mutational status) and to doublet/triplet backbone chemotherapy, the benefit of adjuvant treatment for early and locally advanced CMS4 tumors is not obvious [39,40,41,42]. To date, CMS classification offers richer insights into the molecular heterogeneity of CRC and prognosis, but its role in clinical decision-making is still to be confirmed [36,43].

## 2. Analyzing the Opportunities to Increase Therapeutic Index for CRC by Using DSB Repair Inhibitors

Cancer chemotherapy and radiotherapy are designed to kill cancer cells mostly by inducing DNA damage and disturbing replication or mitotic machinery. DNA-damaging agents cause various types of DNA lesions, including base modification, intrastrand crosslinks, interstrand crosslinks (ICL), DNA–protein crosslinks, single-strand breaks (SSBs), and double-strand breaks (DSBs). The DNA DSB inducing agents are often exploited in cancer treatment, as DSBs cause greatest genomic instability, leading to cell death in absence of functional repair mechanisms. DSBs are mainly repaired by two pathways: homologous recombination (HR) and non-homologous end-joining (NHEJ) repair [44]. However, DNA repair–deficient cancers often become dependent on backup DNA repair mechanisms, such as microhomology-mediated end-joining (MMEJ) in HR deficient background [45].

Despite the comprehensive advances of the CRC molecular landscape, a limited number of biomarkers have made it to the clinic. Thus, most systemic therapeutic options for CRC still rely on chemotherapy-based regimens for early disease, and few targeted agents are recommended for clinical use in the metastatic setting. Moreover, the impact of cytotoxic, targeted agents and immunotherapy combinations in the metastatic scenario is considered suboptimal rather than life changing. Hence, to improve the outcomes for patients who develop resistance to chemotherapy agents and/or are not eligible for targeted agents or ICB, there is an urgent unmet clinical need in the CRC landscape.

Conventional CRC chemotherapy includes combination regimens with 5-FU, oxaliplatin, and irinotecan. The 5-FU-mediated cytotoxicity relies on thymidylate synthase (TS) inhibition, and misincorporation of fluorouracil and uracil into DNA during replication, requesting multiple DNA repair pathways. Incorporation of fluoronucleotides into RNA leads to impairment of RNA processing and function, contributing to the cytotoxic effect [46]. pMMR CRC cells have increased sensitivity to 5-FU in relation to dMMR CRC cells, because of a futile cycle of repair in attempt to remove the misincorporated bases. Base Excision Repair (BER) pathway, acting by uracil–DNA glycosylase (UDG) and AP endonucleases, creates lesions, such as abasic sites, SSBs and DSBs in response to 5-FU [47]. Moreover, 5-FU was shown to cooperate with DSB-induction in CRC cells by decreasing efficiency of HR interfering with the synthesis of new DNA strands [48]. Oxaliplatin toxicity is characterized by the formation of ICLs and intrastrand cross-links. Moreover, platinum drugs induce DNA-protein cross-links that block DNA replication as well [49,50]. The repair of oxaliplatin-induced ICLs involves the FA pathway, which can use parts of the nucleotide excision repair (NER), HR, and translesion synthesis (TLS) [51]. The topoisomerase I poison Irinotecan induces DNA DSBs during S phase and relies on HR for repair [52].

DNA repair deficiency is a frequent event in tumorigenesis, which results in enhanced mutation rates and genetic instability, thus providing a selective growth advantage, a driving force for tumor evolution. Genetic alterations also modify the cellular response to chemotherapy-induced damage. Defects in DNA repair pathways could provide new therapeutic strategies, still not explored in CRC [53]. Moreover, the use of specific DNA repair inhibitors, targeting essential DNA repair pathways, allows us to provoke failures in DNA Damage Response (DDR) of cancer cells. Recent data have demonstrated that prevalence of somatic DDR defects in CRC ranges between 10% and 30%, and predict worse outcomes and resistance to therapy [53,54]. Of all DDR and DNA repair pathways, genes involved with the repair of DNA double-strand breaks (DSBs) are somatically mutated in up to 20% of CRC [54,55]. Fortunately, evolving evidence obtained from large database analyses of CRC tumors have identified DDR-based signatures, which may underpin for the eligibility of treatment with novel (e.g., inhibitors of ATR, CHK1, WEE1, and ATM), and marketed DNA repair inhibitors (e.g., PARP inhibitors), and immunotherapy [56]. Although MMR deficiency remains the unique relevant DDR-based signature for CRC prognosis and response to therapy, the identification of deficiencies in other DNA repair pathways may unveil mutational signatures and provide insights into new targets.

### 2.1. HR-Deficient Phenotypes in Colorectal Cancers

Multiple hereditary or somatic cancers are deficient in homologous recombination (HR) repair, which is the main error-free repair pathway involved in DSBs repair in S and G2 phases and reactivation of blocked replication forks [57]. It has been suggested that a subset of CRC patients (6–15%) harbor mutations in HR genes, including *ATM, BRCA1/2*, *MRE11A*, *FANCC*, *NBN*, *PALB2* [54,55,58,59]. Data retrieved from The Cancer Genome Atlas (TCGA) and other databases have shown that genes critical for HR, such as *ATM*, *BRCA1,* and *BRCA2*, are somatically mutated in more than 20% of CRC [55]. Additionally, 8.7% of colorectal tumors harbor non-silent mutations in gene code regions concomitantly in HR and BER or HR and MMR pathways [60]. More recently, it has been described that patients with brain metastases of primary colorectal tumors exhibit mutational signatures of homologous recombination deficiency (HRD) due to mutations on *BRCA1*, *BRCA2*, *RAD51B*, and *PAXIP1* [61].

Identification of HR deficiency is classically described in tumors with germline mutations in *BRCA1/2,* such as breast and ovarian [62]. In these tumors, HR deficiencies grant higher sensitivity to alkylating or platinum-based agents as a result of generation of non-processed and highly toxic DSBs. Data of a study enrolling 108 stage III CRC patients found a germline mutation followed by *ATM* or *BRCA2* somatic mutations in 13.8% and 22.2%, respectively [63]. Moreover, tumors without functional BRCA1/2 are sensitive to poly ADP-ribose polymerase 1 inhibitors (PARPi) [64]. Although PARP inhibitors were found to be selectively active in a subset of tumors harboring *BRCA1* or *BRCA2* mutations due to the synthetic lethality of PARP inhibition and this particular HRD, it also has a role in *BRCA* wild type cancers [65,66]. However, HR deficiency phenotype can also occur following genetic and epigenetic inactivation of other HR components in sporadic cancer, which leads to the so-called *BRCAness* signature [67]. This is particularly noteworthy in the CRC context because it may expand therapeutic options for those who do not benefit from targeted (*RAS* mutated) or immune therapies (microsatellite stable, MSS). In a recent review article, we discussed the modulation effects of the main DNA repair pathways in the clinical and pathological aspects of CRC and its potential as prognostic and predictive biomarkers [68].

### 2.2. MMR-Deficient Phenotypes in Colorectal Cancers

The occurrence of CRC due to germline mutations in the MMR genes *MLH1*, *MSH2*, *MSH6*, and/or *PMS2* or *EpCAM* represents 2% to 4% of all CRC cases. About 19% of sporadic colorectal tumors may also present somatic MMR defects following acquired mutations or hypermethylation of the *hMLH1* gene promoter [69]. dMMR is clinically equivalent to microsatellite instability-high (MSI-H), whereas pMMR is the same as microsatellite instability-low (MSI-L) or MSS [70]. Impaired MMR function is more frequent in stage II disease (22%) than in stage III disease (12%), whereas in stage IV it occurs in only 3.5% of the cases, suggesting a reduced tendency for distant metastasis [69]. Since discrepancies in the frequency of MMR deficiencies between stages [71], several studies have investigated if this molecular phenotype may be a predictive marker of reduced benefit from adjuvant fluoropyrimidine-based regimens in these settings. Two large retrospective studies have shown that MMR deficiency predicts response to adjuvant 5-FU or capecitabine in these patients [72,73], but more recent studies have failed to prove dMMR as a biomarker of fluoropyrimidines efficacy for stage II disease [74,75]. Therefore, both observation and fluoropyrimidine-based adjuvant chemotherapy are recommended for stage II disease with or without MMR deficiencies and irrespective of recurrence risk. Yet, only for patients at high-risk of recurrence or with dMMR colorectal tumors, adjuvant treatment may include 5-FU/LV or capecitabine in combination with oxaliplatin (FOLFOX, or CAPOX), respectively [36]. In 2021, NCCN recommended the investigation of MMR status for all stages to help with hereditary CRC diagnosis, and in stage II for decision-making if 5-FU-based adjuvant therapy is indicated.

The dMMR sporadic CRC have exclusive features such as delayed onset, frequent *BRAF^V600E^* mutations, and better disease prognosis (hazard ratio for overall survival: 0.59; 95% CI: 0.50–0.69) [76]. Additionally, dMMR colorectal tumors are usually infiltrated by intraepithelial and peritumoral lymphocytes in response to a high production of neoantigens generation following the high mutational burden resultant from MSI [55]. Enriched lymphocytic infiltration is associated with boosted anti-tumor immunity. Therefore, it might explain why MSI is observed more often in stages II and III colorectal tumors rather than in metastatic disease, where tumor cells were able to evade immune surveillance [77]. Most of these tumors present loss of MLH1 and PMS2 proteins due to hypermethylation of the *hMLH1* gene promoter typically in association with the CpG island methylator phenotype (CIMP) [78]. Genes regulating cell proliferation, survival, cell cycle and DNA repair which harbor microsatellites are disposed to mutations due to dMMR [77]. Several studies have shown that oncogenic RAS activation is associated with replication stress, increased HR-mediated response, increased DNA damage levels, and genomic instability [79,80,81]. Recently, the mutational profile of DDR has been described in the largest transcriptomic study in colorectal tumors [82]. This study included 9321 CRC patients and demonstrated that *RAS* and *BRAF* mutational status might have a different impact on the DDR signature in relation to MSI status. From the total, 1290 CRC patients presented mutations in the DDR genes (13.8%) associated with MSI-H/dMMR tumors, with frequency of 76.4% (456/597) vs. 9.5% in MSS/pMMR tumors. The highest mutation frequency between the 29 DDR genes evaluated were detected in *ATM* (4.5%), *BRCA2* (2.7%), *PRKDC* (1.6%), and *CHEK2* (1.2%) genes. The authors have found that *RAS*-mutant tumors had significantly lower frequency of DDR mutations in relation to the *RAS*-wild type tumors. However, there was no significant difference in the subgroups (MSS/pMMR or MSI-H/dMMR). Conversely, the *BRAF*-mutant-type had a significantly higher DDR-mutational profile in comparison to *BRAF*-wild type tumors (31.1% vs. 12.1%). Likewise, no difference was observed in relation to the MMR proficiency. Amongst the 1529 samples available for CMS analysis the frequency of DDR mutations was highest in the CMS1 subtype (34.8%) as compared to the other subtypes (CMS2—7.1%, CMS3—15.2%, and CMS4—11.8%). In *RAS*-mutant tumors, differential lower levels of gene expression were observed in *CDK12*, *NBN*, *BRCA1*, *MRE11A,* and *PRKDC*, whereas regarding *BRAF*-mutant tumors, lower gene expression was observed in *CDK12*, *MRE11A*, *RAD50*, *ATR,* and *FANCF* [82]. Taken together, these observations suggest the distinctive levels of DDR activity according to the gene expression of HR, NHEJ, DNA damage checkpoint, and Fanconi anemia pathways, as well as MSI status may have a role in the response to therapy in individual patients.

Most patients with a classic Lynch syndrome phenotype carry germline mutations in one of the MMR genes, all of them leading to deficient mismatch repair and consequent MSI in the tumor [83]. The MMR proteins Msh2 and Mlh1 were also used by MMEJ pathway implicated in antibody gene class switch recombination [84]. In sporadic CRC, the presence of MMR deficiency (mostly due to epigenetic inactivation of *hMLH1* promoter) is associated with a better prognosis. In this field, our research group has shown that the presence of MSI in sporadic CRC is associated with a reduction in initiation of base excision repair (BER) by the DNA glycosylases OGG1 and MPG, as well a reduction in signaling of DNA single-strand breaks (SSB) repair by PARP1 [85].

The choice of DNA repair pathway determines which type of genomic instability arises from replication stress-associated DNA double-strand breaks [86]. MSI induction in vitro was triggered by replication stress-associated DSBs via DNA repair dependent on both Polθ and PARP1, which is likely mediated by MMEJ in MMR-deficient background [87]. In this scenario, the DSBs are first recognized by HR factors, followed by the HR–MMEJ switch attributed to complex formation. Recent study reported that the MMR complex MSH2-MSH3 at DSB site promotes HR, facilitating long-range resection by EXO1, and inhibits the access of Pol θ [88]. The induction of MSI in MMR-deficient cells is associated with hypermutation phenotype and mutations in cancer-driver genes, resulting in clonal expansion [89]. At the same time that MSI is induced, chromosome instability (CIN) is suppressed in MMR-deficient cells (because of DSB elimination). CIN was proposed to arise when the DSBs are not effectively repaired in S and G2 phases by the HR. Following a defective mitosis, such DNA breaks activate DDR in the G1 phase of the next cell cycle and eventually are ligated to incorrect broken ends by NHEJ, leading to chromosomal alterations [90,91]. Tumors with CIN represent distinct type of genomic instability in CRC (about 60% of all cases), characterized by chromosomal rearrangements and aneuploidy, gene amplifications and deletions, loss of heterozygosity (LOH), and micronuclei [92].

Clinically, mutations in *MRE11* occur in the majority of MMR deficient colorectal tumors [93], and are associated with increased susceptibility to CRC [94]. Moreover, it has been suggested that MSI arises from meiotic recombination 11 homolog (MRE11) deficiency due to defective interactions between MLH1 and MRE11 [95]. While upregulation of MRN complex has been associated with a high tumor grade, chemo/radiotherapy resistance, and poor overall and progression-free survival, deficiency in MRE11 predicts a better prognosis [96,97,98,99]. Conversely, for CRC with functional MMR pathway, high MRN expression is associated with earlier tumor grade and good prognosis [100,101]. Recently, it has been reported that MRE11 is an independent favorable prognosis in left-sided, but no right-sided CRC, which may suggest a novel molecular feature according to the disease laterality [102]. Thus, targeting MRE11 to sensitize colorectal tumor cells considering MSI status may be an interesting approach to overcome resistance and expand antineoplastic combinations. Moreover, CRC cells harboring MRE11 deficiencies are more sensitive to PARP inhibitors [103], platinum salts [97], and 5-fluorouracil/leucovorin/ irinotecan combination [96].

## 3. Cellular Responses to DNA DSB

**Figure 1:**
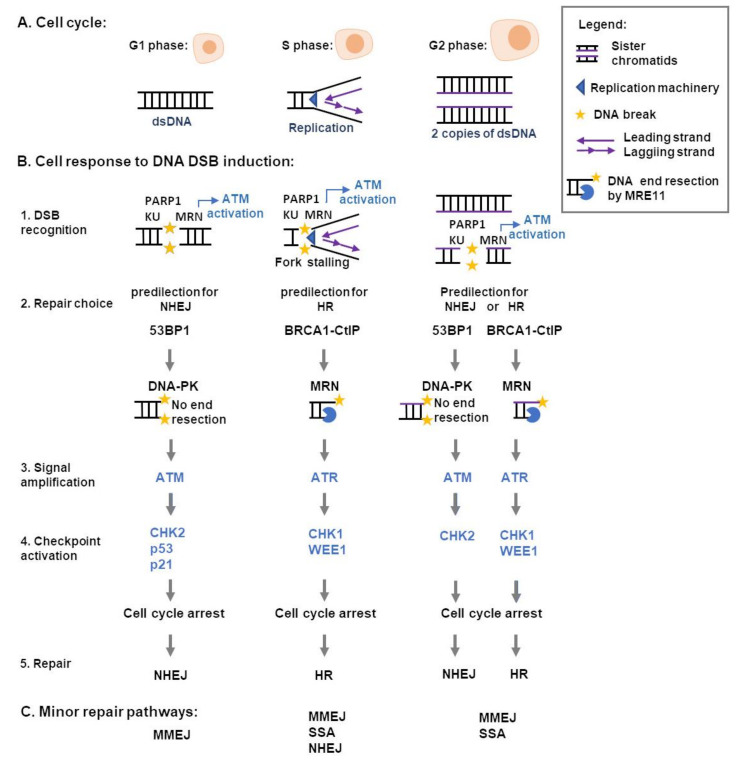
Cell cycle-dependent response to DSB formation. Depending on the stage of cell cycle (**A**), the induction of DSB (**B**) will lead to distinct cellular ATM or ATR signaling responses and DNA repair pathways. While DNA-PK drives NHEJ repair without resection of DNA ends, the MRN-CtIP removes DNA-PK and HR can function and use sister chromatids as a template for DNA repair synthesis. (**C**) Upon some context, including HR defect, the MMEJ and SSA repair pathways can act on the resected single-stranded DNA. MMEJ can act as a backup for NHEJ in G1 phase.

The cell response to DSB formation involves three main steps to enable DNA repair: (1) the recognition of the lesion by sensor proteins (MRE11-RAD50-NBS1 complex, Ku and PARP1); (2) the amplification of the DNA lesion signal by transducer proteins (ATM, ATR, DNA-PKcs) and (3) the cell cycle checkpoint activation by effector kinase proteins (CHK1, CHK2) (Figure 1) [104,105,106].

When DSB are formed, after gamma-ray ionizing radiation for example, there is an increase in the MRE11-RAD50-NBS1 (MRN) complex formation [107]. The MRN complex binds to the DNA ends of the break site and Nijmegen breakage mutated protein 1 (NBS1) interacts with Ataxia-telangiectasia-mutated (ATM) kinase in such a way that ATM is activated by autophosphorylation of Ser 1981 [108]. The ATM activation, in turn, increases the MRN complex formation [107], and initiates a phosphorylation cascade at key proteins: NBS1-Ser343, CHK2-Thr68, and p53-Ser15 [104]. The DNA repair occurs without induction of CHK2-dependent checkpoint when cells present lesions below a certain threshold level; otherwise, CHK2 is autophosphorylated in Thr387, driving p21 accumulation and cell cycle arrest in G1-phase [109,110,111].

In S and G2 phases MRN transiently activates ATM, then an ATM-to-ATR switch is driven by formation of single-strand DNA coated by RPA after MRN resection of DNA ends. Then the ataxia-telangiectasia and Rad3 related (ATR) activation phosphorylates CHK1 that phosphorylates WEE1 and these events promote cell cycle arrest [112,113,114]. The cell cycle arrest in G2-phase can also be maintained by ATM-CHK2 activation at unresected DSB and 53BP1 keeps the ATM activity to phosphorylate CHK2, thus enhancing ATM-CHK2 signaling (Figure 1B) [115].

Besides MRN, the DNA-PK complex—formed by DNA-PKcs and Ku—also binds to DNA ends during all the cell cycle phases within DSB is formed [105,116]. Ku is a heterodimer (Ku70 and Ku80 subunits) that binds DNA end and recognize DSB simultaneously to the MRN complex [105]. Ku recruits the catalytic subunit of DNA-PK (DNA-PKcs) to form the complex named DNA-PK and drives canonical NHEJ repair. Besides Ku and MRN, PARP1 is recruited to DSB and in S/G2 phase, its enzymatic activity has a role to remove Ku from the DSB site [106]. The pathway choice for DSB repair is cell cycle regulated and depends on DNA end resection (Figure 1B). The NHEJ repair occurs throughout G1, S, and G2 phase, and is preferred for repair of two-ended DSBs with compatible ends, while the HR is preferred for the repair of one-ended DSBs, such as replication-associated lesions, which favoring HR in S phase [117]. It is proposed that Ku plays a role in regulation of arrested replication fork restart, where the initial resection by MRN-Ctp1 is essential for recruitment of HR factors in *Schizosaccharomyces pombe.* The lack of Ku leads to reduced recruitment of RPA and Rad51 and extensive resection by Exo1 [118]. Moreover, in human cells, the Ku binds to single-ended DSB, and the formation of the DNA-PK complex is necessary to initiate MRN endonuclease activity [105,119]. During S and G2 phases, CDKs, and other factors that promote DNA end resection are activated, and remove Ku from DNA; hence, NHEJ is favored when the resection is limited, as in G1 phase [117]. In the G2 phase, the cell can choose either pathway; however, complex DNA ends or DSBs in transcriptionally active loci preferentially undergo HR repair [117,120]. In addition to HR and NHEJ, which are the main DSB repair pathways, the error-prone repair pathways microhomology end-joining (MMEJ) and single strand annealing (SSA) also can participate in the DSB repair. Both MMEJ and SSA require a prior DSB end resection mediated by CtIP and MRN leading to loss of genetic information. These pathways play a backup role for the HR, while MMEJ also has a role as backup for the NHEJ (Figure 1C) [121,122].

To drive NHEJ repair, 53BP1 is phosphorylated by ATM, protecting the DNA ends from resection [123]. There are other positive regulators of NHEJ repair downstream 53BP1, like RIF1, REV7, and RINN1/2/3, and they inhibit BRCA1 accumulation at the DSB site [124,125]. When the cell drives HR repair, BRCA1 prevents ATM-dependent 53BP1 phosphorylation [123]. The phosphorylation of CtIP by ATM, ATR, or CDK at specific sites—T847 and T859—promotes BRCA1-CtIP interaction, and CtIP stimulates MRN nuclease activity that generates a single-stranded DNA (ssDNA) [119,125]. Moreover, BRCA1-CtIP recruits DNA2 exonuclease and CDK phosphorylates EXO1, which expand the ssDNA in cooperation with DNA2 exonuclease and BLM helicase, removing DNA-PK from DNA (Figure 1B) [126,127,128].

The nuclease activity of MRE11 protein has also a role in the choice of DNA damage repair pathway: the MRE11 endonuclease activity is the initial step for resection in HR repair forming a single-stranded nick, followed by exonuclease activity that generates a ssDNA gap and so prevents NHEJ repair [129]. The inhibition of MRE11 exonuclease activity inhibits HR without increasing NHEJ repair, while inhibition of the endonuclease activity enhances NHEJ by reducing HR [129,130]. Moreover, at collapsed replication forks the MRN complex structurally assists the linking of the end DNA with the sister chromatid through a RAD50 hook that facilitates the homologous recombination repair [131].

## 4. DNA Replication Fork Arrest, Replication Stress, Checkpoint Activation, and Genome Instability in Cancer

The integrity of the human genome is affected by exogenous insults, such as chemical carcinogens and ionizing radiation, as well as DNA damage that occur during the process of chromosome duplication when the DNA replication forks are slowed down or stalled by varied natural replication barriers, a process referred as replication stress (RS) [132,133]. RS is detected at early stages of carcinogenesis and is considered as a driving power of cancer progression [134,135,136]. RS in cancers frequently results from the oncogene-driven perturbation of the replication initiation program (origin activation and timing) and increased conflicts between replication and transcription. This triggers the generation of under-replicated regions as well as the persistence of stalled and collapsed forks, major sources for chromosomal breakage and chromosome instability [137]. If two converging replication forks stall with no possible compensation by licensed origins in-between, a double fork-stalling event occurs leading to the generation of under-replicated parental DNA and causing chromosome breaks when the cells enter mitosis [137,138]. Collapsed forks lead also to chromosomal breakages and alterations providing a permanent sub-population of cellular variants upon which selection could act, allowing some mutant sub clones to multiply explaining tumor heterogeneity, development, and drug resistance.

While genome instability is associated with poor prognosis, excessive karyotypic instability is deleterious for cancer cell fitness and correlates with improved survival outcome, supporting that a threshold limits extreme risky RS, DSBs, and genome instability in cancers [139]. Consequently, cancer cells need to adapt to the severe replicative defects and the resulting excess DSBs. This is why some of these adaptive mechanisms are currently considered exploited, therapeutically. The first adaptive response to a high level of RS is the ATR-CHK1 checkpoint response, which manages the stability, the repair, and the restart of arrested forks avoiding premature entry into mitosis and allowing the completion of DNA replication [140]. In agreement with the importance of the checkpoint response in cancers for limiting excessive RS, high levels of the checkpoint mediators Chk1, Claspin, and Timeless known to stabilize the stalled replication forks upon RS have been found to correlate with poor patient survival [57,141]. The second relevant adaptive response to RS is the late mitotic DNA synthesis or MiDAS and its critical actor Rad52, a mechanism that differs from semi-conservative DNA replication in S-phase and counteracts lethal chromosome mis-segregation by limiting the persistence of under-replicated DNA in mitosis [138]. As the under-replicated DNA is located ahead of the incoming fork, MiDAS requires replication-coupled repair named break-induced replication (BIR) [142,143]. The third category corresponds to a specialized DNA repair process, an alternative form of end-joining, referred to as microhomology-mediated end-joining (MMEJ; also named polymerase theta-mediated end joining, TMEJ) that substitutes for HR deficiency for the repair of DSBs generated by excessive RS and for which the A-family DNA polymerases Pol θ, encoded by the gene *POLQ*, holds a key role.

## 5. Emerging DSB Repair-Targeting Therapies for Colorectal Cancer

Most of the in vitro studies with potential inhibitors that may improve CRC therapy consider biomarkers to predict response. Although some DNA repair inhibitors have not been explored in CRC cell lines, there is scientific basis to be applied (Table 1).

### 5.1. Homologous Recombination Repair

**Figure 2:**
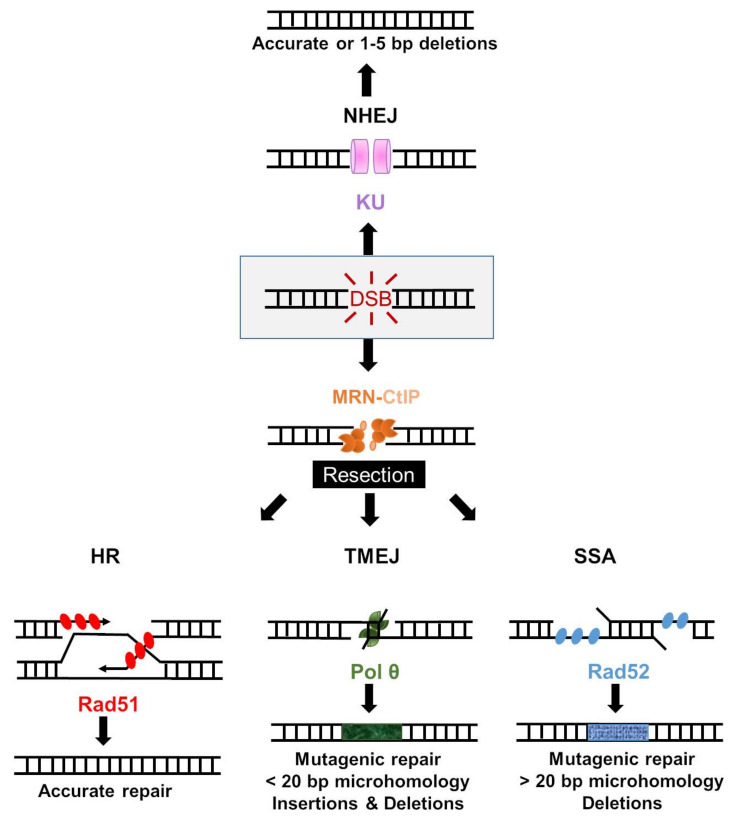
Major double-strand break (DSB) repair pathways in human cells. DSBs in G1 of the cell cycle are primarily repaired by non-homologous end joining (NHEJ) (Top). This pathway is initiated by the Ku heterodimer, which recognizes broken DNA. NHEJ leads to repair with minimal alteration to the original sequence. DSBs in S/G2 phases are subjected to resection leading to stretches of single-stranded DNA. Resected DSBs are substrates for homologous recombination (HR) with a critical role of Rad51 leading to an accurate repair synthesis. When HR is defective (for example when BRCA genes are mutated), an alternative pathway named Pol theta-mediated end-joining (TMEJ) can act as a back-up repair pathway. Polθ promotes the synapsis of the opposing ends, identifies internal microhomologies, which can be annealed, and performs a repair DNA synthesis with poor processivity and frequent aborted synthesis, resulting in a high rate of mutations. Single-strand annealing (SSA) is a HR sub-pathway in mammalian cells with the essential role of RAD52 DNA-binding protein. SSA is an error-prone repair leading to large deletions between the homologous repeats.

Double-strand breaks formed during the S and G2 phases of cell cycle, where the sister chromatids have been generated, are repaired mainly by the homologous recombination (HR) pathway [159]. In contrast to NHEJ, MMEJ, or SSA, HR is a conservative pathway, which uses the intact sister chromatid as a template and, therefore, restores the DNA sequence without loss of genetic information (Figure 2) [160,161].

HR starts when the MRN complex recognizes the breaks; MRE11 initiates the DNA end resection to produce ssDNA that is then covered by RPA, and the recombinase RAD51 assembles onto ssDNA after RPA displacement [162]. The formation of such RAD51-ssDNA filament is essential for the search of the homologous sequence and its invasion through the formation of a D-loop intermediate to ensure the repair DNA synthesis [163]. It has been reported that expression of the HR proteins MRE11 and RAD51, monitored by immunohistochemistry predicts the response and prognosis of colorectal cancer patients who received oxaliplatin chemotherapy [97]. A significantly better tumor reduction and longer PFS was observed in MRE11- and RAD51-negative cases compared with MRE11 or RAD51 positive cases, suggesting that inhibition of these two main HR actors could improve sensitivity to chemotherapy [97]. In the same way, *ATM* and *BRCA2* somatic mutations are suggested to be biomarkers that predict response to stage III CRC patients that received oxaliplatin-based chemotherapy, and they are associated with recurrence-free survival [63].

#### 5.1.1. Targeting MRE11 in CRC

Genetic instability in MSI tumors, known to result from variation in microsatellite tracts due to a defective MMR process, can be further enhanced by mutations in microsatellite tract repeats in the *MRE11* gene [164]. A microsatellite tract of 11(T) located at intron 4 of *MRE11* is mutated in approximately 80% of MSI tumors and leads to aberrant splicing and a truncated protein inhibition of MRE11 nuclease activity. Importantly, such mutated *MRE11* gene has been shown to sensitize CRC cells to agents causing DNA replication inhibition [165], as well as PARP-1 inhibition [101,103], suggesting that targeting MRE11 in MSS CRC might be an interesting therapeutic strategy. Inhibition of MRE11 by mirin, an inhibitor of its exonuclease activity discovered in 2008 [166], showed promising results in killing myeloma and neuroblastoma cell lines displaying high level of replication stress and endogenous DNA damages, evidenced by high amount of γ-H2AX and RAD51 foci as well high rate of ATR phosphorylation. Enhanced sensitivity of these cells seems specific to HR since they were also sensitive to the RAD51 inhibitor B02, but not the DNA-PK inhibitor NU7441 [154]. Such an effect of mirin was also observed in neuroblastoma cells with high degree of replicative stress. The MRN complex was shown to be important to prevent replicative stress (RS) generated by MYCN during the expansion of cerebellar granule progenitor cells [167]. In some tumor cells, *MYCN* oncogene is amplified, which increases RS levels [168], and is considered a bad prognosis in neuroblastoma [169]. However, it was observed that MYCN is a transcriptional regulator of MRE11, RAD50, and NBS1 [167], and the MRN complex contributes to the control of DNA damage levels compatible with tumor cell survival, so that high MRE11 expression is related to reduced overall survival in primary human neuroblastoma [155]. The use of MRE11 exonuclease inhibitor mirin increased the RS and DNA damage biomarkers in *MYCN*-amplified neuroblastoma cells until death in vitro and induced apoptosis in vivo. The inhibitor presented a promising outcome by reducing tumor growth in neuroblastoma-xenografted mice treated for 11 days with encapsulated mirin compared to control [155]. These results suggest that mirin should be explored in other tumors with high RS levels as well. Primary cancer stem cells from colorectal cancer (CRC-SC) isolated from patient samples showed adaptive response to high levels of RS induced by CHK1 inhibition. CRC-SC that were resistant to CHK1i showed PARP-1 upregulation, which decelerated fork progression and decreased RS levels. The combined inhibition of MRE11 and RAD51, by mirin and B02 co-treatment, selectively killed PARP-1-upregulated CRC-SC via mitotic catastrophe [156]. These data suggest that MRE11 and RAD51 cooperate with PARP-1 for RS response. There are other MRE11 inhibitors besides mirin that inhibit endo- (PFM01 and PFM03) or exonuclease (PFM39) activities [129]. However, to date, none is under investigation in clinical trials for CRC patients.

The alternative MMEJ pathway to repair DSBs operates on a common resected HR intermediate and includes the factors involved in the 5′ to 3′ resection (e.g., MRE11, RAD50, NBS1, CtIP, and EXO1). MMEJ requires PARP1 in its early step and starts with the search of short tracts of contiguous microhomology, followed by an annealing step, and then by a processing mechanism of 3′ ssDNA tails and DNA synthesis ensured by the specialized DNA polymerase Pol θ, a major mediator of this pathway [170,171]. MMEJ contributes to 10–20% of DSB repair in mammalian cells, is independent of γ-H2AX signaling, and seems to be poorly requested at the G0 and G1 phases of the cell cycle while it is activated upon S-phase entry [172]. The absence of MRN, the use of mirin, or the expression of the MRE11-H129N nuclease mutant impairs HR and MMEJ to a similar extent [172,173,174].

On the other hand, the use of mirin could also be explored in association with anti-PD-L1 therapy. T lymphocytes can recognize foreign antigens; however, CRC cells with MSI-H or dMMR tumors can upregulate immune checkpoint proteins, such as PD-1 and PD-L1, which permit immune evasion. Currently immunotherapy based on checkpoints inhibition in advanced CRC is limited to MSI-H tumors and other biomarkers being investigated [175]. Both MRN and PD-L1 overexpression are associated with platinum-based chemotherapy resistance [98,176,177]. Recently an association was discovered between PD-L1 and the MRN complex through an immunoprecipitation technique. It was found an interaction of PD-L1 with NBS1, the protein responsible for supporting the MRN complex and for ATM activation. The silencing of NBS1 or PD-L1 sensitized cells resistant to cisplatin, but the silencing of both proteins together showed synergism and induced 80% of cell death; and the synergism was confirmed in vivo [178]. It is known that the ATM/ATR activation induces PD-L1 expression [179], which is one of the reasons that cells with DNA damage repair deficiency respond to PD-L1 blockade [180,181], besides neoantigens formation [182,183]. However, ATM is activated by the MRN complex, so inhibiting the complex could help to inhibit ATM activation, thus explaining the synergism between anti-PD-L1 and NBS1 silencing. The ATM activation does not depend on MRE11 nuclease activity; however, it was shown that mirin inhibits ATM activation by some other way not yet understood [166,184]. Therefore, considering the multiple possibilities of mirin usage, including inhibition of HR repair, MMEJ and ATM activation, this compound could bring beneficial outcomes in CRC or other tumors. It can be employed alone or in combination with other therapies, such as anti-PD-L1 and PARP inhibitors, or in conditions of increased endogenous-DSB formation caused by replicative stress.

#### 5.1.2. RAD51 Inhibition

The inhibition of RAD51 is also utilized as a strategy to impair HR repair in cancer treatments wherein this pathway is important for cell survival. RAD51 participates in HR repair, which is the preferable choice for DSB repair as it is the only one usually error-free pathway [161]. RAD51 also has a role in protection of stalled replication forks by covering the nascent DNA. FANCI-FANCD2 complex directly binds to RAD51 to stabilize the RAD51-ssDNA filament, thus it prevents RAD51 dissociation from DNA and protects DNA end from FEN1, DNA2 and MRE11 nuclease degradation [185,186,187]. RAD51 also enables fork regression by a switch in template strand of stalled replication fork, so that DNA polymerase can bypass the DNA lesion on the leading strand template that is blocking replication fork progression [188]. Thus, the nascent DNA of the opposite branch is used as a template for extension of the leading strand and fork restoration forming a “chicken-foot” structure [189]. The fork regression does not depend on the RAD51 enzymatic function [186], but depends on the proliferating cell nuclear antigen (PCNA) poly-ubiquitination [190,191]. Some compounds focus on inhibiting the RAD51 strand invasion catalysis (via D-loop formation) of HR repair without affecting the ssDNA binding activity of RAD51, which is important for protecting stalled replication forks [192]. The RAD51 mRNA levels analyzed in CRC samples from 48 patients undergoing surgery and without preoperative chemotherapy were upregulated in 2.5-fold compared to non-tumor tissue and associated with T stage [193]. Despite the absence of independent prognostic value of RAD51 mRNA levels in patients without undergoing chemotherapy, in another study, RAD51 expression predicted the response and prognosis of patients who received oxaliplatin chemotherapy; thus, RAD51 may be a candidate for targeted chemotherapies [97,193].

The emergence of RAD51 inhibitors has enabled new therapeutic approaches that target HR repair in vitro, such as in *KRAS*-mutant CRC cells. Colorectal cancers show a high frequency of activating *KRAS* mutations [194], and it was found that *KRAS*-mutated HCT116 cell line (constitutively active *KRAS*) shows increased stalled replication forks, pRPA32 (S33), and γ-H2AX signaling, besides the increase in RAD51 expression compared with the isogenic wild type cell line (HKe-3); all these signals together suggest a dependency on HR repair [150]. Then Kalimutho et al., (2017) wondered whether RAD51 could be a molecular target to impair HR repair and sensitize CRC cells to death induction. Indeed, the depletion of RAD51 by siRNA in *KRAS*-mutant HCT116 cell line decreases cell survival without decreasing survival of WT cells, as well as RAD51 inhibition by RI-1 inhibitor [150]. In another study, it was also suggested a CRC dependency on HR repair as it was found a rise in expression levels of HR proteins RAD51 and BRCA2 in CRC biopsy in comparison with normal mucosa biopsies [48]. Interestingly the inhibition of RAD51 in the SW480 CRC cell line by B02 treatment was enough to induce apoptosis. It was not even necessary to induce DSB before B02 treatment to sensitize the cells, indicating a potential therapeutic approach [48]. Besides IR-1 and B02, there are other RAD51 inhibitors yet to be explored in CRC: RI-2, CYT01B, IRB2, and RAD51-IN-2.

### 5.2. Alternative End-Joining

The alternative end-joining (Alt-EJ) is represented by a variety of repair pathways, genetically different from NHEJ, that repair DSBs by initial end resection generating 3′ single strands. These pathways are intrinsically mutagenic and play an important role in DSBs repair in cancer cells generating deletions and genomic rearrangements [195]. Alt-EJ events start with limited resection by MRN-CtIP, such as HR, and were almost entirely abolished by inhibition of the resection with MRE11i [196]. The Alt-EJ can be classified in relation to the repair proteins that promote the pathway, or by the characteristics of the repair junctions with different mechanisms of formation proposed (summarized in recent review) [197]. It is an independent, tightly regulated pathway that operates, even when the HR and NHEJ are functional. Alt-EJ is active predominantly in S and G2 phase but it also occurs in G1 phase [198,199]. The repair of DNA DSBs by Alt-EJ can be mediated by microhomologies (i.e., MMEJ), typically <20 bp in mammalian cells [195,200]. There is also poorly defined Alt-EJ that can be independent of sequence homology [195]. DNA polymerase theta (Pol θ) is a predominant mediator of the MMEJ pathway (named TMEJ), that generates short insertions and deletions (Figure 2), associated with specific mutational signatures in human cancers [196,201,202].

#### 5.2.1. Polymerase Theta-Mediated End Joining

Microhomology-mediated end-joining (MMEJ), or polymerase theta-mediated end joining (TMEJ), is used at a substantial frequency to repair DSBs in cycling cells even when both NHEJ and HR pathways are available [172]. Unlike HR, MMEJ is intrinsically error-prone and produces deletions and translocations (Figure 2). In mammalian cells, MRE11 nuclease, CtIP, DNA ligase III, and an error-prone multifunctional DNA polymerase theta (Polθ, encoded by the *POLQ* gene) have critical roles in MMEJ [203]. In the first step of MMEJ in human cells, PARP1 binds to the DSB ends and facilitates the recruitment of the resection factors, CtIP, and MRN complex generating ssDNA and allowing microhomology search. The microhomology annealing induces formation of non-homologous 3′ ends that can be eliminated by Polθ prior to DNA synthesis as recently reported [204], suggesting that Polθ can itself remodel DNA ends to facilitate their repair. The tetrameric Polθ-helicase domain prepares the substrate by microhomology alignment, followed by filling-in synthesis by the Polθ-polymerase domain and ligation by LigIII. Polθ–helicase domain is essential for efficient joining of DNA breaks, acting to dissociate RPA (that prevents spontaneous annealing), and promote the annealing of complementary DNA [205].

#### 5.2.2. Polymerase Theta (Polθ) Inhibition

POLQ was first reported as a powerful biomarker in several solid cancers, where its overexpression correlated significantly to poor patient survival [206]. Later, Polθ has emerged as a promising target for the treatment of various DNA repair–deficient cancers, including those that are deficient in HR [170,207]. Cancers with upregulation of MMEJ pathway display a distinctive mutational signature and present new targets for cancer therapy [208]. The HR factors BRCA1 and BRCA2 play a role in suppressing MMEJ in human cells [209], and BRCA2 protects stalled replication forks from degradation [210]. The depletion of these proteins increased break-induced mutational frequencies and short microhomologies at the break-site. The observed mutational signature may arise from HR failure at a stage after the initiation of resection, and was dependent on the MMEJ factors—CtIP, PARP1, MRE11, and Polθ. Moreover, in this context, the repair may occur in Polθ-dependent MMEJ and possibly by Polθ-independent microhomology-mediated pathways suppressed by RPA [209]. The Polθ-dependent pathway, also called TMEJ, generates unique short flanking DNA microhomologies, which Polθ efficiently locates when present or creates de novo when absent [211].

Interestingly, *POLQ* expression is increased in cancers, both with and without HR deficiency [212,213]. Moreover, Polθ is coping with replication stress and repairing DSBs upon fork collapse [212,214]. In HR-proficient background, inactivation of *POLQ* increased cell death in ATR-deficient cells and sensitized breast cancer cells overexpressing *POLQ* to DNA damaging agents that cause replication stress (camptothecin and etoposide) or fork collapse (ATR inhibitors) [214]. *POLQ* expression in different cancer types strongly correlated with the expression of factors involved in response to replicative stress (*RAD51*, *FANCD2,* and *BLM*), instead of genes encoding for several core MMEJ proteins, factors involved in DNA end resection or in canonical NHEJ [212].

There is plenty of evidence that deficient DNA repair pathways (HR, MMR, and BER) lead to genomic instability. Several studies have indicated that Polθ may have an important role in limiting excess genomic instability and allowing tumor cell survival when exposed to oncogenic-replicative stress, or when canonical error-free repair pathways are impaired [207,215,216,217]. In fact, the overexpression of *POLQ* in cases of colorectal, gastric, lung and breast cancers are associated with a worse overall survival [206,218,219].

Genomic sequencing of breast cancer and colorectal adenocarcinomas has identified specific mutations and microhomologies at the rearrangement breakpoints, suggesting involvement of MMEJ [220,221]. Whole-genome sequencing of 2559 samples from 38 tumor types showed that the junction rearrangement could be generated by different formation mechanisms, i.e., NHEJ (no sequence homology at the breakpoint junction), MMEJ (2–7 bp of microhomology) and SSA or others, including microhomology-mediated BIR (10–30 bp of microhomology) [222]. Beyond the NHEJ, synthesis-dependent MMEJ can also form simple deletions and apparent blunt-end junctions [197]. Between those samples, data from 52 colorectal adenocarcinomas showed elevated heterogeneity and high rates of the fragile-site signature [222]. Studies of breakpoint sequences across different tumor types indicated that 27–50% of the genomic rearrangements could be attributed to microhomology-mediated mechanisms [223]. In some tumor types, the breakpoint microhomology has been associated with genomic stability and tumor invasiveness. For example, invasive bladder tumors are characterized by genetic instability and microhomology at rearrangement breakpoints (indication of MMEJ activity), whereas non-invasive tumors do not show microhomology and are genetically stable with error-free DNA repair profile [224].

Moreover, *POLQ* is largely absent in most normal tissues, representing a promising tumor-specific target for cancer treatment. In this regard, knockdown of *POLQ* increased the sensitivity of a panel of tumor cell lines from different primary sites to radiation in contrast to the normal tissue cells [225]. *POLQ* was revealed to be synthetic lethal with DNA repair genes frequently mutated in cancer (such as *ATM*, *ATR*, *BRCA1/2, FANCD2*, *Ku70*, *RAD52*, *RAD51C*, *TP53BP1*) and extensive efforts for the development of Polθ inhibitors are made by several companies [226]. The antibiotic Novobiocin was shown to inhibit Polθ, thus decreasing viability of HR-deficient tumor cells due to accumulation of toxic RAD51 foci [158]. As HR repair and MMEJ shared a common resection step with participation of MRE11 nuclease [172], Polθ inhibitors could strongly synergize with MRE11 inhibitors. Polθ inhibitors could also synergize with PARPi in HR-deficient cancers and possibly overcome acquired resistance to monotherapy [207,227].

### 5.3. Single-Strand Annealing

Single Strand Annealing (SSA) is an important DSB repair pathway, which rely on annealing of homologous repeats flanking a DSB, generating loss of genetic information by large deletions (up to several hundred bp) between the repeats (Figure 2) [228]. It was shown that chromosomal translocations at Alu elements, the most numerous family between the repetitive elements that comprise nearly half of the human genome, occur predominantly by SSA [229]. The complementary DNA sequences involved in SSA are more than 20–25 nucleotides that distinguish it from the MMEJ [195]. PARP-1 mediates the recruitment of MRN and CtIP to the DSB for end resection, which is an essential step of SSA. The resultant short single-strand region is rapidly coated by RPA and serves as a binding site for the processive Exo1 or DNA2 exonucleases [195,228]. The resultant long-range resection exposes complementary regions with more than 25 nucleotides. The next steps are annealing by the Rad52 protein that has a robust single annealing activity on single strand complementary sequences, and removal of the non-homologous 3′ DNA tails by ERCC1/XPF complex. After these, the gaps are filled by DNA polymerases and ligated by DNA ligases that are not well defined for SSA.

#### RAD52 Inhibition

Human RAD52 was recently revealed as a promising candidate for targeted therapy in relation to its important role in replication fork metabolism, promoting cellular viability in cancer cells [230]. RAD52 is a DNA-binding protein involved in the HR sub-pathway single-strand annealing (SSA) in mammalian cells. SSA is an error-prone repair leading to deletions between the homologous repeats, thus increasing genomic instability. Synthetic lethality studies have shown that, in the absence of BRCA2, BRCA1, and PALB2, RAD52 is essential for DSB repair [231,232,233]. The RAD52 N-terminal domain was shown to be essential for maintaining viability of BRCA-deficient cells promoting DSB repair by homology-directed repair (HDR) and SSA [234,235]. The activity of RAD52 responsible for HDR in these cells relies on D-loop formation rather than mediation of RAD51 loading on single-stranded DNA in the presence of RPA. RAD52 plays a role also in replication forks repair and mitotic DNA synthesis (MiDAS) upon replication stress [138,142,230]. Mammalian RAD52 participates in break-induced replication (BIR) repair of collapsed DNA replication forks in cancer cells [236].

RAD52 foci co-localized with FANCD2 into mitotic chromatin following replicative stress (RS), together with RPA and MUS81 [138]. Since RS is the main mechanism of genomic instability in cancer cells, it is extensively studied for identification of targets in cancer therapy. The persistence of stalled forks can lead to fork collapse and chromosome instability (CIN), which is generally associated with poor prognosis, promoting tumor heterogeneity and drug resistance. High RAD52 protein expression in tumor samples from 179 patients who underwent surgery for rectal cancer was associated with worse disease-free survival [233]. None of the cases with RAD52 protein expression was classified as microsatellite instability (MSI)-high (MMR-negative).

Currently there is an increasing clinical interest in the use of RAD52 inhibitors (in addition to inhibitors of PARP, Chk1, and Polθ) in order to exacerbate the RS in human cancers [237]. RAD52 has a limited role in DNA repair of normal cells like Polθ, thus providing a selective therapeutic target. Small-molecule RAD52 inhibitors are discovered by structure-based selection and proposed to be suitable for disruption of RAD52 rings in BRCA-deficient cancers [238]. RAD52 inactivation increased cell death in lung tumors and in BRCA2-deficient cancer cells [138]. Combined disruption of RAD52 and POLQ conferred synthetic reduction in the velocity of replication fork restart and had additive effect on cisplatin toxicity [239]. Moreover, combination of RAD52 and PARP1 inhibitors could improve the response to treatment in HR-deficient cancers [238,240].

### 5.4. Cell Cycle Checkpoint Inhibition

The cell cycle checkpoint can be impaired by inhibiting the ATM-CHK2-p53 pathway or the ATR-CHK1-WEE1 pathway. Cells that lack functional p53 depend on the G2 checkpoint for DNA repair and survival since the G1 checkpoint is activated by the p53-p21 pathway that is defective in these cells [241]. Inhibition of the G2 checkpoint in p53-deficient cells has shown to increase the sensitivity to some DNA damage inducing agents in vitro [144,151]. Interestingly, there is a high frequency of *TP53* mutation in CRC samples. The germline, followed by *TP53* somatic mutation (single-nucleotide variants) was found in 70.4% of stage III CRC patients [63]. Moreover, data from 50 CRC patients showed *ATR* and *CHK1* expression significantly increased in the tumor, compared to adjacent mucosa, and *ATR* expression has significantly increased in the late stages (III and IV), thus *ATR* and *CHK1* appear to be important for tumor progression [242].

#### 5.4.1. ATR Inhibition

In vitro studies indicate that ATR inhibitors can be used in combination with oxaliplatin, cisplatin, or CHK1 inhibitor to inhibit the proliferation of colorectal cancer cells. The inhibition of Nbs1-dependent ATR activation in p53-deficient colorectal cancer cell (HT29) increased sensitivity to oxaliplatin and cisplatin chemotherapeutic agents through a reduction in proliferation, increase in sub-G1 cell population and cleaved-caspase3 after 72 h treatment with the inhibitor CBP-93872 [151]. In this study, the ATR inhibitor suppressed the G2 checkpoint induced by oxaliplatin and cisplatin, as well as suppressed the downstream CHK1 activation [151]. Once these cells are p53-deficient, they depend on G2-checkpoint for survival, which can be suppressed by ATR-CHK1 signaling inhibitors. The ATR inhibition also showed an increase in cytotoxicity when combined with a CHK1 inhibitor in CRC cells in another study. The co-treatment prevented ATR-dependent feedback activation of CHK1 and hence increased the replication stress of cancer cells [144]. The results of this study showed that the CHK1 inhibitor V158411 decreased cell proliferation and induced DNA damage in the CRC cells HT29 and U2OS, assessed by nuclear staining for γ-H2AX, pCHK1 (S317), pCHK2 (T68), and pRPA32 (S4/S8). Moreover, the addition of ATR inhibitor VX-970 with the CHK1 inhibitor increased the DNA damage and the cell growth inhibition in a time and dose dependent manner [144]. The combination of ATR and CHK1 inhibitor reinforce the suppression of checkpoint, which can be lethal for cells undergoing replication stress.

#### 5.4.2. CHK1 Inhibition

The induction of DSB needs to be repaired to cell survival; hence, cell promotes cell cycle arrest. However, cells that lack p53 depend on ATR-CHK1 checkpoint, thus inhibiting this axis is an attractive strategy. In fact, the inhibition of CHK1 by prexasertib (LY2606368) killed primary CRC enriched for cancer stem cells (CRC-SC). However, not all samples showed sensitivity. When they correlated sensitivity with genome sequencing found that *TP53* mutations were a biomarker predicting sensitivity, as well as phosphorylation of ATM or RPA32—replicative stress markers—and increased chromosome number. CRC-SC with these markers depend on CHK1 activity, so LY2606368 treatment by inhibiting CHK1 impaired cell cycle checkpoint resulting in lethal replication catastrophe [145]. Interestingly, the inhibition of RAD51 (B02) or MRE11 (Mirin) was able to sensitized resistant CRC-SC to prexasertib by induction of replication stress, while others inhibitors of DDR proteins ATM, ATR, or DNA-PK were ineffective [243]. RAD51 and MRE11 are HR factors, the main pathway activated for resolving DSB during replication, and CRC-SC are tolerable to high replication stress level due to modulation of DNA damage response [156]. These data evidence RAD51 and MRE11 as key regulators of CHK1-resistant CRC-SC survival and support the future development of clinical trials with these treatment regimens.

#### 5.4.3. ATM Inhibition

The ATM-p53 pathway is considered one of the major synthetically lethal partners. *ATM* is mutated in ~20% of colorectal cancers, which are mostly missense mutations and scattered throughout the coding region [244]. However, functional effects of the *ATM* mutations are not clear; ataxia telangiectasia (A–T) mutations in *ATM* are known to induce protein truncation, protein destabilization, and resulting loss of function [245]. In metastatic CRC patients, 15% harbor somatic *ATM* mutations, which was associated with improved overall survival. Considering CRC patients with *TP53* mutations, 69% have a co-mutation in *ATM* [58]. It was recently reported that 13.8% and 22.2% of stage III CRC patients harbor somatic mutations in *ATM* or *BRCA2*, respectively. Moreover, patients carrying *ATM*, *BRCA2,* or non-*TP53* APC somatic mutations treated with oxaliplatin-based chemotherapy had a better outcome than those without such mutations [63].

The in vitro studies indicate that the ATM inhibition sensitizes CRC cells to chemotherapy, and the p53 status is involved in ATM inhibitor sensitivity [152]. A variety of ATM-deficient cancer cell lines showed sensitivity to PARP inhibitors, including CRC cell lines. Considering this evidence, the use of ATM inhibitor could sensitize cancer cells to the PARP inhibitor Olaparib as well [152,246]; so, the ATM inhibitor KU55933 was tested in the ATM-proficient HCT116 cell line and sensitized it to PARP-inhibitor Olaparib, decreasing the colony formation number after 14 days and the depletion of p53 enhanced the sensitivity [152]. In another study, ATM inhibitor (AZ31) also enhanced the antiproliferative effect in CRC cell lines (HCT15, HCT116, and RKO) and patient derived xenografts with the addition of topoisomerase I inhibitor (SN38), due to a cytostatic effect (increase in G2/M cell cycle arrest without apoptosis induction) [153]. It was found that cell lines sensitive to the co-treatment presented in an intriguing way no p53 protein levels, despite showing increase in p53 phosphorylation (s15) levels after the co-treatment with the ATM inhibitor and irinotecan, while resistant cells presented p53 protein levels and showed little increase in P-p53 (s15) levels after the co-treatment. Interestingly the PDX models that showed sensitivity to combined treatment were irinotecan resistant, while the irinotecan sensitive had little sensitivity to the co-treatment, yet additional study is necessary to identify irinotecan resistance biomarkers [153]. Using samples from metastatic CCR patients the ATM-loss was not associated with improvement in overall survival after irinotecan-based therapy, but after oxaliplatin-based therapy. However, the p53 levels were not evaluated in this study [247]. Notably the use of checkpoint inhibitors depends on the expression of cell damage response biomarkers as well as the damage inducing agents applied.

#### 5.4.4. WEE1 Inhibition

The WEE1 upregulation in colorectal tumor samples has been reported, although whether this upregulation has prognostic value or not remains controversial. Upregulation of WEE1 is suggested as a potential prognostic biomarker for CRC patients [248]. The WEE1 mRNA levels were increased in tumor tissues and the positive staining of WEE1 was found in the most of 102 CRC tissue samples. Both results were correlated with distant metastasis and high TNM stage [248]. In another study, the checkpoint kinase WEE1 was highly expressed in primary CRC obtained from patients of a prospective cohort but it did not reach independent prognostic value [249]. Moreover, an in vitro study has pointed out that WEE1 inhibition selectively kills tumor cells, so WEE1 inhibitors may have a role as targeted therapy for CRC. The inhibitor adavosertib (AZD1775, MK-1755) induced apoptosis in CRC liver metastases endothelial cells (CLMECs) isolated from patients. The WEE1 inhibition had preferential effects on CLMECs than on matched normal liver endothelial cells because CLMECs had WEE1 mRNA and protein levels upregulated in comparison to the normal cells [146]. This particular WEE1 inhibitor also increased 5-FU cytotoxicity in *TP53*-mutated CRC cell line (HT29) by induction of DSB and apoptosis [147]. Moreover, adavosertib decreased cell survival of CRC cell line (HCT116) in combination to PARG inhibitor (PDDX-004/PDD00017272) in vitro, and in PARG knockout cell clones (HCT116 PARG KO) in xenograft model by induction of DNA damage in S-phase [148]. Adavosertib was also reported to increase apoptosis of the *TP53*-mutated CRC cell line (HT29 and SW480), and sensitize the cells to irinotecan. These effects may have occurred due to inhibition of the G2 checkpoint so the cell loses the ability to repair the DNA damage induced by irinotecan [149]. The p53-status may be a biomarker and could be considered in clinical trials.

## 6. Clinical Trials of DDR Inhibitors in CRC Patients

In colorectal tumors with DDR-deficient background, combinations of cytotoxic agents and DSB repair-targeting therapies (e.g., PARP, ATM, ATR, or CHK1inhibitors) may be useful. The studies with checkpoint inhibitors are more advanced than those with DNA repair inhibitors, as some of them have already reached clinical trials (Table 2).

There are currently four candidates in clinical trials acting as ATR-kinase inhibitors—berzosertib, ceralasertib, elimusertib, and M4344. Of these, berzosertib and ceralasertib are the most studied candidates. Although more than 50 clinical trials in phase I/II have been investigating these ATR inhibitors, only one study including CRC patients has published their results so far. The phase I clinical trial of berzosertib in monotherapy or in association with carboplatin included 11 patients with *KRAS* and *BRAF* wild type advanced CRC harboring ATM loss and an AT-rich interactive domain 1A (*ARID1A*) mutation (NCT02157792). Of the three CRC patients treated with monotherapy, one achieved stable disease with an ongoing progression-free survival of 29 months. Baseline tumor analyses of this patients revealed complete absence of ATM protein expression, although there was no evidence of ATM mutation, MMR deficiency (loss of MLH1 and PMS2) and truncating mutations in several DNA repair enzymes, including two heterozygous truncating mutations in ARID1A and heterozygous truncating mutations in *CHEK1*, *RAD50*, *POLD1* [256].

*ARID1A* is commonly mutated in CRC (9.4%), which results in loss of protein expression with consequent impairment of SWI/SNF chromatin remodeling complex. In CRC, *ARID1A* mutation frequency is enriched in tumors with MSI and loss of ARID1A in CRC with late TNM stage, poor pathological classification, and distant metastasis [262,263]. Recently, epigenetic regulator and DNA repair proteins, having synthetic lethality interaction with *ARID1A* mutation, have been reported, such as inhibition of PARP and ATR. In ARID1A-deficient tumors, decreased accessibility of 53BP1 to DNA lesions leads to reduced NHEJ activity, rendering high sensitivity to PARP inhibitor therapy after exposure to exogenously induced DNA breaks such as ionizing radiation [264]. Moreover, CRC cell lines harboring *KRAS* mutations are critically dependent on ARID1A function [265]. Interestingly, evidence exists that both NHEJ and HR can be affected by the depletion of the other subunit of the SWI/SNF complex, ARID1B, in ARID1A-deficient cells. A recently study found that an impaired interaction between these two members of SWI/SNF complex and HR enzymes in CRC *ARID1A*-mutated cell lines contribute to an increased radiosensitivity and reduced RAD51 foci induction, indicating reduced homologous recombination [266]. Taken together, these findings may open up perspectives for new therapeutic combinations aiming to disrupt DSB repair in CRC without an HRD background.

CHK1 inhibitors have a long history in clinical trials, however, their use is associated with elevated toxicity, especially for the low selectivity first-generation CHK1i [267]. From seven CHK1 inhibitors that entered in clinical trials, only three remain active: prexasertib, LY2880070, and SRA737. The phase I/Ib clinical trials of prexasertib for patients with advanced or metastatic solid tumors included CRC patients with *KRAS* and/or *BRAF* mutations (NCT02860780) and CRC patients with *KRAS* wild type CRC who has failed to oxaliplatin- and irinotecan-based chemotherapy (NCT02124148) to evaluate safety of the combinations of prexasertib with ralimetinib or cetuximab, respectively [250]. However, in the study of prexasertib + ralimetinib (a p38 mitogen-activated protein kinase inhibitor), the recommended phase 2 dose (RP2D) of prexasertib were not established due to dose-limiting toxicities [250]. The LY2880070 safety and efficacy of LY2880070 in monotherapy or in combination with gemcitabine (NCT02632448) is under investigation in a phase IB/IIA for solid tumors, including CRC.

The WEE1 inhibitor adavosertib has been under investigation in phases I/II since 2008, and currently, there are 28 clinical trials with this molecule still active. The combination of adavosertib + gemcitabine + cisplatin or carboplatin was assessed in a phase I trial for several solid advanced tumors (NCT00648648). Partial response and stable disease lasting at least 6 weeks as best overall response were observed in 10% and 53% of the patients, respectively. Sixteen patients (8%) presented advanced or metastatic CRC, and one patient had stable disease [260]. A new WEE1 inhibitor, ZN-c3, is under investigation in a phase I/II clinical trial for solid tumors, including CRC (NCT04158336). It has recently been reported that five patients reached stable disease and two had partial response (ovarian cancer and CRC patients). Moreover, a 42% reduction in overall target lesions was observed in CRC patients, which remained about 6 months on study drug before disease progression [261].

Despite CRC high incidence, prevalence and understanding of its molecular etiology, disease staging and tailored therapeutic approaches are still limited. Even for late-stage disease, to which systemic treatment options are biomarker-driven, resistance inevitably occurs [268]. Within this scenario, downregulation of MMR (MLH1 and MLH6) and HR genes (BRCA1/2, RAD51) and upregulation of error-prone DNA polymerases (e.g., Pol iota, Pol kappa) have been implicated as a key mechanism of acquired-resistance to targeted-therapies [269]. A recent study with CRC cell lines and patient-derived xenograft models treated with cetuximab or an anti-BRAF (dabrafenib) has shown that CRC cells can evade targeted therapies by switching off DNA repair pathways, particularly through downregulation of HR. These cells demonstrated compromised DNA repair and persistent elevated DNA damage levels. This observation may reveal another opportunity to elucidate whether anti-VEGF/EGFR-resistant CRC cells may be sensitized by agents, such as PARP inhibitors or the novel agents targeting the ATR-CHK1-WEE1 axis [269].

## 7. Molecular Selection of CRC Patients for Clinical Trials with DDR Inhibitors

**Figure 3:**
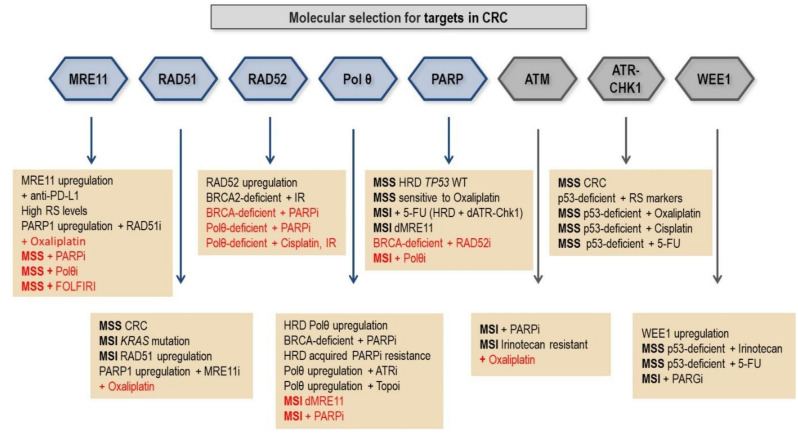
Possible targets for DDR inhibitors (repair proteins in blue and checkpoint kinases in grey) in CRC chemotherapy with supporting evidence in cancer cell lines and animal models, or candidates “to be tested” (in red), with estimated sensitivity based on results obtained in cells with deficiency of the respective target. HRD—homologous recombination deficiency, IR—ionizing radiation, Topoi—topoisomerase inhibitor, MMS—microsatellite stable, MSI—microsatellite instable, RS—replication stress, dATR-Chk1—deficient ATR-Chk1 signaling, FOLFIRI—folinic acid/fluorouracil/irinotecan chemotherapy regimen.

Although DNA repair genes alterations and the resulting genomic instability have been explored in other tumor types, only few DDR inhibitors were employed as monotherapy or combined with chemotherapy in trials enrolling CRC patients, including PARP, WEE1, ATR, and CHK1 inhibitors. Two recent review articles summarizing the ongoing and completed clinical trials concluded that the obtained unsatisfactory results in some trials could be attributed to a lack of molecular selection of patients to be included for any specific treatment [53,270]. Moreover, the lack of characterization of functional repair deficiencies and consensus on the defined set of DDR genes to be tested were appointed as limiting factors. A growing body of evidence suggests that the sensitivity of CRC cells and tumors to therapy induced DNA damage and repair inhibition could differ in relation to the microsatellite instability status and functionality of HR (summarized on Figure 3). Only in one trial with published results—the microsatellite status was used as criteria to stratify 33 patients with disseminated colorectal cancer (20 MSS and 13 MSI-H) enrolled for Olaparib testing, where no statistical difference was found in the median progression-free survival and overall survival between the two groups [271]. However, in this study Olaparib was used as a single-agent and not in combination with DNA damage inducing chemotherapy or radiation therapy. In this respect, in vitro studies have shown a synergistic effect of Olaparib and 5-FU in MSI-H CRC [272], whereas Olaparib monotherapy showed benefits in MSS CRC with non-functional HR, and was proposed as maintenance therapy in patients responsive to oxaliplatin-based regimens [273]. The enrichment of PARPi sensitive MSS cell lines was observed in those with preserved TP53 function and TP53-mediated suppression of RAD51 was appointed as a possible mechanism of action [274]. Further, data from 99 MSS CRC cell lines revealed that functionality of HR repair determined in the RAD51 assay could discriminate for PARPi susceptible CRC tumors, in which no increase in the percentage of RAD51 foci positive nuclei was observed upon radiation [273]. Moreover, this study did not show association between the sensitivity to the PARPi Olaparib and mutational signatures correlated to HR defects or *BRCAness*, as well as to specific CMS or mutations in *KRAS* and *BRAF*. RAD51-low score was found as an indicator of response (to PARP inhibitors and carboplatin) and patient outcome in two studies reported on ESMO Breast Cancer Virtual Congress 2021, and incorporation of RAD51 testing for clinical decision making in triple negative breast cancer was proposed [275,276].

In order to assess the tumor HR status, a combined HRD score was proposed, calculated as unweighted sum of three independent scores for estimation of genomic instability: telomeric allelic imbalance (TAI), loss of heterozygosity (LOH), and large-scale state transitions (LST), that can be determined on a pretreatment biopsy [277]. Using this DNA-based measure, the authors found that tumors with HR deficiency, with a threshold of HRD score ≥ 42 for breast and ovarian cancers, were responsive to neoadjuvant platinum-based therapy. Recent study extended the utility of HRD analysis for sensitivity to agents inducing RS and DNA double-strand breaks (such as 5-FU, oxaliplatin, irinotecan, topoisomerase inhibitors, between others) to all solid cancers from The Cancer Genome Atlas and Cancer Cell Line Encyclopedia [278]. The authors showed that the HRD cases with history of treatment with DNA-damaging agents (*n* = 2979) had better overall survival than treated non-HRD cases, while in absence of treatment the HRD cases (*n* = 6460) had a worse outcome than non-HRD cases. Moreover, the HRD score was higher in tumors with *TP53* mutations. The results of this study suggested that personalization of the treatment based on HRD status as a therapeutic biomarker has a potential to improve the precision and efficacy of chemotherapy irrespective of the cancer type [278].

## 8. Conclusions

In spite of advances in identification of clinical and molecular features with significant prognostic and predictive value in CRC, the therapeutic options still mostly rely on conventional chemotherapy-based regimens rather than in targeted therapies, in particular regarding early stages. Chemotherapy is considered a “one-size-fits-all” approach, which often results in resistance to treatment and suboptimal outcomes. Targeting genes involved in the responses to replication stress and DNA DSBs repair, such as *MRE11*, *RAD51, RAD52,* and *POLQ*, alone or in combination with DNA damaging agents and checkpoint inhibitors, might be a strategy to expand the spectrum of systemic therapeutic options, thus tailoring CRC treatments and improving the disease prognosis. Moreover, molecular selection of alterations in DNA repair genes, and the resulting genomic instability for targeted DDR therapy in sub-populations of CRC patients could be helpful because of tumor heterogeneity. An increasing body of evidence suggests that the sensitivity of CRC cells and tumors to DNA damage, inducing chemotherapy and DNA repair inhibition, could differ in relation to the microsatellite instability status and functionality of HR. Current clinical testing of CRC includes characterization of the microsatellite status, which is considered for selection of chemotherapy regimens and for inclusion in clinical trials, along with mutations in oncogenic driver genes (*BRAF*, *KRAS*, etc.), but alterations in DNA DSB repair genes are not investigated. However, recent work has identified additional genomic (i.e., HRD score) and functional assays of DNA repair (i.e., RAD51 assay) that provide new predictive and pharmacodynamic biomarkers for targeted DDR therapies.

## Figures and Tables

**Table 1:** Proteins related to replicative stress and DSB processing: situation of inhibitor studies (in vitro) and relevant biomarkers.

Inhibitor	Biomarkers	Combined Therapy or Deficiency	Model of Study	Results	Biological Explanation	Reference
Inhibitors in perspective for clinical trial in CRC (inhibitors already tested in clinical trial for another tumor type or condition)						
PARPi (ABT-888)	Presence of MSI	MRE11 deficiency	Homozygous *MRE11* mutant CRC cell lines (HCT116, LoVo, RKO, SW48) and wild type cell lines (HCT15, SW403, HT29, SW620)	Cell lines with homozygous *MRE11* mutation had increased sensitivity to PARPi.	PARPi induces DSB during replication, which is repaired mainly by HR repair. However, the presence of MSI is associated with HR impairment by MRE11 deficiency, which sensitizes cells to PARPi.	[101]
ATRi (VX-970)	-	CHK1i (V158411)	CRC cell line (HT29 and U2OS)	Both inhibitors induced loss of viability and the combination of them in low doses increased sensitivity.	Both proteins act on the same cell cycle control axis-repair of DNA DSB forming during S and G2 phases. The inhibition of both reinforces the blocking of the axis, generating replicative stress.	[144]
CHK1i (LY2606368)	*TP53* mutation and RS markers	-	Primary CRC enriched for cancer stem cells	Sensitive cells displayed signs of replicative stress (P-RPA32, γ-H2AX, and P-ATM) and the effect did not depend on RAS mutation status.	Cells that lack p53 depend on the ATR-CHK1 axis to promote cell cycle arrest when DSB is formed. Therefore, the inhibition of CHK1 kinase in these cells sensitizes them to DSB. Replicative stress induces DSB, therefore cells ongoing RS are sensitive to CHK1i.	[145]
WEE1i (AZD1775)	WEE1 overexpression	-	Primary CRC liver metastases endothelial cells	Induction of apoptosis, increase in DNA DSB markers and inhibition of tube formation.	The WEE1 protein is upregulated in CRC liver metastases compared to endothelial cells of the normal adjacent liver and has functional importance by protecting against DNA DSB. The inhibition of WEE1 makes cells vulnerable to DSB.	[146]
WEE1i (AZD1775)	*TP53* mutation	5-FU	CRC cell line (HT29)	The WEE1i single treatment had cytotoxic effects by induction of DNA DSB. The co-treatment with 5-FU increased DSB marker (γ-H2AX) and apoptosis compared to the 5-FU single treatment.	The lack of functional p53 prevents G1 arrest, so the cell depends on the G2 checkpoint to repair DNA damage. WEE1 is a kinase that regulates the G2 checkpoint to repair DNA damage, so targeting WEE1 increases DNA damage sensitivity.	[147]
WEE1i (AZD1775)	-	PARGi (PDDX-004/PDD00017272)	CRC cell line (HCT116)	The co-treatment increased DNA DSB marker (γ-H2AX) in S-phase dependent manner.	WEE1i induces replication stress and DNA damage while PARGi delays the replication fork restart during replication stress. The combination of both increases DNA damage.	[148]
WEE1i (MK1775)	*TP53* mutation	Irinotecan	CRC cell line (HT29 and SW480)	The co-treatment increased DNA DSB marker (γ-H2AX) and apoptosis compared to single treatments.	The lack of functional p53 prevents G1 arrest, so the cell depends on the G2 checkpoint to repair DNA damage. WEE1 is a kinase that regulates the G2 checkpoint to repair DNA damage, so targeting WEE1 increases DNA damage sensitivity.	[149]
Inhibitors in progress in CRC in vitro studies						
PARPi (LT-626)	Presence of MSI	MRE11 deficiency	CRC cell line with a biallelic mutation in MRE11 (HCT116, HCT116/CS, HCT116/C3, RKO, SW48, and LoVo), with monoallelic mutation (DLD1), and wild type (SW837, HT29, and SW480)	Cell lines with a biallelic mutation in *MRE11* showed higher sensitivity to PARPi and the knocked-down of MRE11 increased sensitivity to PARPi.	PARPi induces DNA DSB during replication, which is repaired mainly by HR repair. However, the presence of MSI is associated with HR impairment by MRE11 deficiency, which sensitizes cells to PARPi.	[103]
RAD51i (RI-1)	*KRAS* mutation	-	CRC cell line (HCT116, HKe-3)	*KRAS*-mutant cell (HCT116) was more sensitive to RI-1 than the isogenic wild type HKe-3, which showed a limited response.	*KRAS*-mutated CRC cells show stalling of the replication fork, which increases HR repair signaling. This occurs because of the hyperactivation of c-MYC. Targeting RAD51, a key protein in HR repair, thus may sensitize *KRAS*-mutated CRC cells.	[150]
RAD51i (B02)	-	-	CRC cell line (SW480)	Induction of apoptosis.	RAD51 expression levels are upregulated in biopsy samples of CRC and may thus support cancer progression.	[48]
ATRi (CBP-93872)	*TP53* mutation	Oxaliplatin, cisplatin, and 5-FU	CRC cell line (HT29)	ATRi single treatment had no effect, however, the combination with compounds that induce DNA damage as DNA DSB (oxaliplatin and cisplatin) or replication fork arrest (5-FU) induced an increase in apoptosis compared to single therapies.	The lack of functional p53 prevents G1 arrest, so the cell depends on the G2 checkpoint to repair DNA damage. ATR activation regulates the G2 checkpoint to repair DNA damage, so targeting ATR activation may increase DNA damage sensitivity by suppressing the maintenance of the G2 checkpoint.	[151]
ATMi (KU55933)	-	PARPi (Olaparib)	CRC cell line (HCT116)	ATMi sensitizes cells to PAPRi and the deletion of p53 increased the co-treatment effect.	PARP1 induces DNA DSB during replication, which activates ATM and so ATR promotes cell cycle arrest and DNA repair. The sensitivity to PARPi increases when the cell lacks ATM levels.	[152]
ATMi (AZ31)	-	Irinotecan	CRC cell line (HCT15, HCT116, RKO, LoVo, LS132, and Caco2) and patient-derived xenografts (PDX)	Three (HCT15, HCT116, and RKO) of the six cell lines presented combinational sensitivity to AZ31 and irinotecan compared to single treatments. In CRC PDX models, the co-treatment was effective only in irinotecan-resistant tumors.	Irinotecan induces DNA DSB, which activates ATM to promote cell cycle arrest and DNA repair. The inhibition of ATM may sensitize cells to DNA DSB.	[153]
Inhibitors in perspective for in vitro analysis in CRC						
MRE11i (mirin)	RS markers	-	Human myeloma cell lines (MM1S, RPMI-8226, JJN3, U266) and B-cell (LINF903).	Only cells presenting RS markers (RAD51 and γ-H2AX signaling) showed sensitivity to mirin.	Human myeloma cell lines presenting DNA DSB signaling depend on DSB repair pathways for survival. To inhibit DSB repair may sensitize them.	[154]
MRE11i (mirin)	*MYCN* amplified	-	Neuroblastoma cell line with *MYCN*-amplification (SHEP, GIMEN, and SK-N-SH) and *MYCN* single copy (LAN5, IMR32, and KELLY) and LAN5 xenograft in mice.	Only the cell lines with *MYCN*-amplification showed increased mRNA levels of MRE11 and sensitivity to mirin treatment. Mirin suppressed tumor growth in xenografted mice.	MRE11 is required to restrain replication stress induced by *MYCN*-amplification. MRE11 inhibition may trigger intolerable levels of RS.	[155]
MRE11i (mirin)	PARP1 upregulation	RAD51i (B02)	CRC-stem cells with upregulation of PARP1, generated by CHK1i (prexasertib) treatment until acquired resistance.	The single treatments with Mirin or B02 were ineffective against CHK1i-resistant cells, but co-treatment killed the cells by induction of mitotic catastrophe and apoptosis.	CHK1i-resistant cells upregulate PARP1 to modulate fork speed and decrease RS levels. MRE11 and RAD51 may cooperate with PARP1 to deal with RS.	[156]
RAD52i (F79 aptamer, D-I03)	BRCA1 deficiency	PARPi (talazoparib)	BRCA1-deficient primary acute myeloid leukemia (AML) xenograft in NSG mice, and BRCA1-deficient solid tumor growth in nude mice.	The combination of PARP inhibitor with RAD52 inhibitors selectively reduced BRCA1-deficient tumor growth.	PARPi induces DNA DSB during replication, which is repaired mainly by HR repair. However, BRCA-deficient cells have HR repair impairment, and single-strand annealing (SSA) is a backup pathway that may support survival. The RAD52 inhibition may sensitize cells to PARPi by the accumulation of DNA DSB in BRCA-deficient tumor cells.	[157]
RAD52i (F79 aptamer, 6-OH-Dopa, D-I03)	BRCA deficiency	PARPi (olaparib, talazoparib)	Several human tumor cell lines	The combination of PARP inhibitors with RAD52 inhibitors selectively killed BRCA-deficient cells.	See description above.	[157]
Polθi (Novobiocin)	BRCA deficiency	PARPi (Olaparib)	Xenograft mice derived from patients with germline *BRCA1* mutation and acquired PARPi resistance, and HR-proficient PDX model.	Olaparib single treatment did not reduce tumor growth, while Polθi single treatment reduced tumor growth and the combined therapy was even more efficient. BRCA1 wild type PDX model was resistant to both single and co-treatment. Polθi toxicity depends on the accumulation of RAD51 foci.	PARPi induces DNA DSB during replication, which is repaired mainly by HR repair, but also by MMEJ repair. BRCA-deficient cells have HR repair impairment and respond to PARPi. However, MMEJ has emerged as a backup pathway in PARPi resistant cells. The Polθ inhibition may prevent MMEJ repair and increase PARPi sensitivity in BRCA-deficient tumor cells.	[158]

MSI: microsatellite instability; CRC: colorectal cancer; DSB: double-strand break; HR: homologous recombination; 5-FU: 5-fluorouracil.

**Table 2:** Clinical Trials of DDR inhibitors in CRC patients.

Inhibitor		NCT Number	Conditions	Primary and Secondary Endpoints	Intervention/Treatment for CRC	Phase(s)	Status	Reference
Chk1 Inhibitor	Prexasertib (LY2606368)	NCT02860780	Advanced/metastatic cancer, including CRC with *KRAS* and/or *BRAF* mutations	MTD	Prexasertib + ralimetinib	Phase 1	Completed	[250]
		NCT02124148	Advanced/metastatic cancer, including *KRAS* wild type CRC, which has failed to oxaliplatin- and irinotecan-based chemotherapy or is intolerant of irinotecan or oxaliplatin		Prexasertib + cetuximab	Phase 1b	Completed	-
	LY2880070	NCT02632448	Solid tumors, including CRC	MTD	LY2880070 ± gemcitabine	Phase 1b/2a	Recruiting	[251]
	SRA737	NCT02797964	Advanced solid tumors (including CRC) and non-Hodgkin’s lymphoma	Subjects with TRAE, MTD, recommended Phase 2 dose, ORR	SRA737	Phase 1/2	Completed	[252]
ATM Inhibitor	AZD0156	NCT02588105	Advanced solid tumors, including CRC	Subjects with TRAE	AZD0156 + irinotecan/ FOLFIRI	Phase 1	Active, not recruiting	[253]
ATR Inhibitor	Ceralasertib (AZD6738)	NCT04704661	Advanced solid tumors, including CRC that have a change (mutation) in the HER2 gene or protein	RP2D, PD profile of tumor tissues between Top1 inhibition and Top1 + ATR dual inhibition	Ceralasertib + trastuzumab deruxtecan	Phase1/1b	Not yet recruiting	-
	Elimusertib (BAY 1895344)	NCT04535401	Advanced or metastatic cancers of the stomach and intestines, including CRC, which have previously progressed on irinotecan with and without DDR defects	MTD	Elimusertib + FOLFIRI	Phase 1	Not yet recruiting	[254]
	Berzosertib (M6620, VX-970)	NCT02157792	Advanced solid tumors, including CRC harboring molecular aberrations, including ATM loss and an *ARID1A* mutation, achieved complete response, and maintained this response, with a progression-free survival of 29 months at last assessment	Safety (AE, laboratory values, ECG), ORR	M6620 + carboplatin	Phase 1	Completed	[255,256]
WEE1 inhibitor	Adavosertib (AZD1775, MK-1755)	NCT02906059	Metastatic CRC with *RAS* (*KRAS* or *NRAS*) or *BRAF* mutated	DLT and TREA	AZD1775 + irinotecan	Phase Ib	Completed	[257]
		NCT02465060	Advanced refractory solid tumors (including CRC), lymphomas, or multiple myeloma	ORR	Adavosertib + targeted therapy according to mutational status	Phase II	Recruiting	[258,259]
		NCT00648648	Advanced solid tumors, including CRC	DLT, best ORR	Adavosertib + gemcitabine + cisplatin or carboplatin	Phase 1	Completed	[260]
	ZN-c3	NCT04158336	Solid tumors, including CRC	MTD, RP2D, DLR, ORR	ZN-c3	Phase I/II	Recruiting	[261]

CRC, colorectal cancer; MTD: maximum tolerated dose; TRAE: treatment-related adverse event; RP2D: recommended phase 2 dose; ORR: objective response rate; PD: pharmacodynamics; ECG: electrocardiogram; SD: stable disease, DLT: dose limiting toxicities; pCR: pathological clinical response; cCR: clinical complete response; PR: pathological response.
